# *Fasciola hepatica*-*Pseudosuccinea columella* interaction: effect of increasing parasite doses, successive exposures and geographical origin on the infection outcome of susceptible and naturally-resistant snails from Cuba

**DOI:** 10.1186/s13071-018-3155-3

**Published:** 2018-10-25

**Authors:** Annia Alba, Antonio A. Vázquez, Jorge Sánchez, David Duval, Hilda M. Hernández, Emeline Sabourin, Marion Vittecoq, Sylvie Hurtrez-Boussés, Benjamin Gourbal

**Affiliations:** 1 Centro de Investigaciones, Diagnóstico y Referencia, Instituto de Medicina Tropical “Pedro Kourí”, La Habana, Cuba; 2 University of Perpignan Via Domitia, Interactions Hosts Pathogens Environments UMR 5244, CNRS, IFREMER, Univ. Montpellier, F-66860 Perpignan, France; 3 Centre de recherche de la Tour du Valat, Arles, France; 4 MIVEGEC, IRD, CNRS, Univ. Montpellier, Montpellier, France

**Keywords:** Snail-trematode interaction, Lymnaeidae, Liver fluke, Experimental infection, Immune priming, Compatibility, Allopatric parasites

## Abstract

**Background:**

*Pseudosuccinea columella* is one of the most widespread vectors of *Fasciola hepatica*, a globally distributed trematode that affects humans, livestock and wildlife. The exclusive occurrence in Cuba of susceptible and naturally-resistant populations to *F. hepatica* within this snail species, offers a fascinating model for evolutionary biology, health sciences and vector control strategies. In particular, resistance in *P. columella* is characterized by the encapsulation of the parasite by host’s immune cells and has been experimentally tested using different Cuban *F. hepatica* isolates with no records of successful infection. Here, we aimed to explore for the first time, the effect of different parasite doses, successive exposures and different parasite origins on the infection outcomes of the two phenotypes of *P. columella* occurring in Cuba.

**Methods:**

To increase the chances for *F. hepatica* to establish, we challenged Cuban *P. columella* with increasing single parasite doses of 5, 15 or 30 miracidia and serial exposures (three-times) of 5 miracidia using a sympatric *F. hepatica* isolate from Cuba, previously characterized by microsatellite markers. Additionally, we exposed the snails to *F. hepatica* from different geographical origins (i.e. Dominican Republic and France). Parasite prevalence, redial burden and survival of snails were recorded at 25 days post-exposure.

**Results:**

No parasite development was noted in snails from the resistant populations independent of the experimental approach. Contrastingly, an overall increase in prevalence and redial burden was observed in susceptible snails when infected with high miracidia doses and after serial exposures. Significant differences in redial burden between single 15 miracidia and serial 3 × 5 miracidia infected snails suggest that immune priming potentially occurs in susceptible *P. columella*. Compatibility differences of allopatric (Caribbean *vs* European) *F. hepatica* with susceptible snails were related to the geographical scale of the combinations.

**Conclusions:**

Here, the effectiveness of *P. columella* resistance to *F. hepatica* does not decline with increasing parasite doses, successive infection or different geographical origins of parasite isolates, while presenting new evidence for specificity for infection in susceptible *P. columella* snails. Understanding the peculiarities of the *P. columella*-*F. hepatica* interaction and the extent of the resistant phenotype is crucial for an effective parasite control and for developing alternatives to tackle fasciolosis transmission.

## Background

The liver fluke *Fasciola hepatica* Linnaeus, 1758 is the main causative agent of fasciolosis, a snail-borne parasitic disease that affects humans, livestock and wildlife [1]. The occurrence of this trematode in all continents except in Antarctica has been largely explained by the introduction of infected livestock and susceptible snails into new areas, with *F. hepatica* being able to parasitize a wide range of host species [1, 2]. The arrival of the liver fluke to the Americas presumably occurred during the early events of colonization of the New World by Europeans, with several native freshwater lymnaeid snails transmitting the parasite today [1].

*Pseudosuccinea columella* (Say, 1817), considered native from North America [3], can be also cited among the intermediate host species of *F. hepatica* in South America and the Caribbean [4–6]. In addition, it is a globally invasive freshwater snail that has been largely introduced out of its native range [3] with reports of established populations from Europe [7], Africa [8], Australia [9] and the Pacific islands [10, 11]. The global spread of some invasive genotypes of *P. columella* might complicate the epidemiological scenario of fasciolosis transmission [3].

Interestingly, in Cuba, *P. columella* displays two different phenotypes regarding *F. hepatica* infection with natural populations being either susceptible [5] or resistant to the liver fluke [12]. Notably, the resistant phenotype in field-occurring *P. columella* is characterized by the encapsulation of the parasite by the host’s immune cells [12]. These populations have been extensively tested for infection using different Cuban isolates of *F. hepatica* but no successful parasite development has ever been recorded (see [12–14] for details). From a genetic perspective, studies exploring the existence of polymorphism in *P. columella* have shown that resistant snails cluster separately from susceptible populations in Cuba [13] and other regions of the world [3].

The challenging of resistant individuals of *P. columella* has been always carried out using a constant standard dose of five miracidia (infective larva for the snails). Therefore, we wanted to explore the effect of higher infective doses of the parasite, either by single or serial exposure trials on both susceptible and resistant *P. columella* using a known polymorphic *F. hepatica* isolate from Cuba (La Palma; [15]). With these approaches we aimed at tipping the scales in favour of the infection success by (i) increasing the probability of encounter of compatible host-parasite genotypes, and (ii) by circumventing or hijacking the effectiveness of host immune defences with large miracidia numbers and/or enhanced genetic diversity of the parasite at which each snail is confronted [16, 17].

In another experiment, given that resistant individuals had always been challenged with Cuban isolates of *F. hepatica*, we exposed for the first time, susceptible and resistant *P. columella* from Cuba to two allopatric liver fluke isolates from the Caribbean (short distance) and Europe (large distance), and compared their infection outcomes with those of the sympatric Cuban isolate. The theory of local adaptation predicts that parasites perform better on their local (sympatric) hosts rather than foreign (allopatric) hosts [18]. However, exceptions exist (e.g. [14, 19]), thus a differential exposure of a host population to an “unknown” entity (i.e. allopatric parasites) might result in differential outcomes and can test for different patterns of susceptibility or even resistance. Given the geographical isolation between the parasite isolates used, genetic differences are expected and should account for variations in compatibility with the snail host, particularly between the Cuban and the European isolates. With this experimental approach, it is likely that a higher infection success from exposing susceptible *P. columella* to sympatric *F. hepatica* would be observed, while the resulting outcome with resistant populations could give clues concerning the specificity of their resistance. If no infection occurs in resistant *P. columella* then we might hypothesize that this phenotypic response is not restricted only to local (Cuban) parasites but it has a broader or even global scale.

Here, we gain new insights on the susceptible *P. columella*-*F. hepatica* interaction, presenting evidence related to differences in compatibility and immune priming as factors affecting the infection outcomes (e.g. prevalence, redial burden, host survival) in this model. Moreover, we demonstrate that resistant populations described from Cuba remain resistant to a high *F. hepatica* miracidial dose, after serial exposures or challenged with allopatric *F. hepatica* isolates. This highly resistant phenotype opened new perspectives of applications for fasciolosis control.

## Methods

### *Fasciola hepatica* isolates and laboratory-reared susceptible and resistant *P. columella*

We collected adults of *F. hepatica* in local abattoirs from eastern Cuba (sympatric isolate: La Palma, Pinar del Río Province), north-western Dominican Republic (allopatric isolate, narrow scale: Dajabón Province) and southern France (allopatric isolate, large scale: Camargue region). The genetic structure of eight *F. hepatica* isolates from Cuba (including parasites from La Palma and other regions within its vicinity) had been previously characterized [15]; this study demonstrated the existence of high polymorphism and genetic diversity within Cuban *F. hepatica* with no clear genetic differentiation among isolates due to a high genetic flow within the island and a preferential out-crossing as reproduction strategy. In particular, the La Palma isolate (local isolate used in the present study) showed over 75% prevalence in sacrificed bovines and a mean of five alleles per analysed microsatellite locus with an observed heterozygosity of 0.511 [15]. Unfortunately, no previous data on genetic diversity is available for the Dominican and French isolates used, but we can expect differences with Cuban flukes given the geographical isolation.

Flukes were collected from the liver of infected cattle and kept alive for 6 h in a solution of 0.85% NaCl (saline solution) and 5% glucose (Sigma-Aldrich, St. Quentin Fallavier, France) for egg laying. Eggs were preserved at 4 °C in the dark and in saline solution supplemented with gentamicin (Sigma-Aldrich, 40 mg/ml) until use.

We used two susceptible *P. columella* populations, Negrines (Havana Province) and Aurora (Mayabeque Province), and two resistant populations, La Coca (Havana Province) and La Palma (Pinar del Río Province). Snails were reared in the Laboratory of Malacology of the Institute of Tropical Medicine “Pedro Kourí”, Cuba, for two to three generations and derived from field-collected populations of *P. columella*. Snails were cultured in Petri dishes with growing algae, in 26 °C de-chlorinated water complemented with crushed shells as a carbonate supplement as previously described [20]. The snails were fed on the algae *ad libitum* and routinely changed to other Petri dishes with growing algae to avoid starvation.

### Experimental exposure of *P. columella* snails to *F. hepatica*

**Fig. 1:**
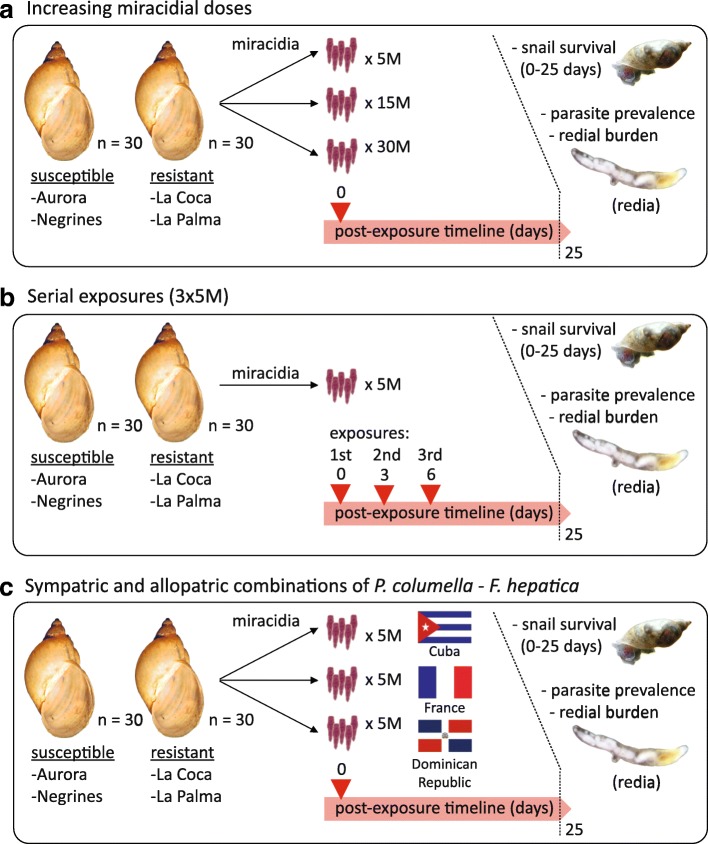
Work flow diagram of the experimental exposures performed on *P. columella* resistant/susceptible-*Fasciola hepatica* model. The influence of different parasite doses (**a**) and serial parasite exposures (**b**) on *P. columella* phenotypes were assayed using sympatric (Cuban) *F. hepatica*. **c** Allopatric *F. hepatica* exposures

Experimental exposure of 2-weeks-old laboratory-reared *P. columella* from the four populations (two resistant and two susceptible) were carried out using freshly hatched miracidia, according to the methodology described by Vázquez et al. [14]. Briefly, eggs of *F. hepatica* were incubated in distilled water in total darkness at 28 °C for 15 days to complete maturation. Miracidia hatching was induced by direct exposure of eggs to light. For each assay, we always used 30 individuals of *P. columella* at varying conditions (see Fig. 1) as described below.

#### A. Exposure to different doses of *F. hepatica *miracidia

We exposed each *P. columella* population separately to single doses of 5, 15 and 30 miracidia (M) from the sympatric isolate of La Palma (Cuba) to increase the probability of exposing host populations to different parasite genotypes.

#### B. Serial exposure to *F. hepatica *miracidia

We explored the effect of serial infections with *F. hepatica* by exposing each *P. columella* population three times to the standard dose of 5 M [14] of the sympatric *F. hepatica* isolate of La Palma (Cuba; 3 × 5 M). Each exposure was performed at a three-day interval, after which each snail received a serially-delivered 15 M dose. The selection of the re-infection interval was based on the timing at which the immune response occurs (0 to 3–4 days post-exposure with patent parasite encapsulation as early as 24 h post-exposure; see Gutiérrez et al. [12]), which is also accompanied by possible “consumption” of defence related resources in the snail.

#### C. Exposure to sympatric and allopatric *F. hepatica*

We exposed each *P. columella* population separately to the standard dose of 5 M [14] of each allopatric *F. hepatica* isolate (Camargue, France and Dajabón, Dominican Republic). In order to save biological material, we used the results of the 5 M dose (see A above), for comparison against sympatric interactions.

In all trials and control groups, snails were individually allocated in 96-well plates and individually exposed overnight to *F. hepatica* miracidia. Each well was checked to record the penetration of all miracidia into the snails used for each experiment. Snails were maintained at 28 °C and monitored daily. Day-to-day mortality was recorded and exposed snails found dead were carefully dissected [21] to assess infection. Between days 7–10 post-exposure, infection was clearly patent by the presence of rediae in dissected individuals. The number of rediae per infected snail was counted to estimate the intensity of *F. hepatica* infection (redial burden) [14] per experimental group, always 25 days post-exposure or post-first-exposure in the case of serial exposure trials. Non-exposed snails from the same breeding batch were reared in the laboratory and subjected to the same conditions to serve as control of survival for the infection assays.

### Data analysis

The prevalence of *F. hepatica* in each experimental group was expressed as the percentage of infected snails of all those initially exposed (*n* = 30); confidence limits were calculated at the 95% confidence level by the Wilson score interval. Differences in prevalence between groups were checked by Fisher’s exact test. Survival curves of exposed snails [from 0–25 days post-(first-)exposure] were constructed based on the number of live snails at each time point divided by the number of exposed individuals and expressed as a percentage. We performed log-rank tests of Kaplan-Meier curves to assess the statistical differences on survivorship data. Data of *F. hepatica* redial counting per experimental group was checked for normality and variance homogeneity using Shapiro-Wilk and Levene tests, respectively. Factorial ANOVAs followed by a *post-hoc* multiple comparison Tukey test were carried out to assess statistical significance of the effect of (i) different miracidial doses and serial exposures within host populations, and (ii) allopatric and sympatric (data of experimental infection with Cuban *F. hepatica* at a 5 M dose) in redial burden. All calculations were performed in Statistica v.12 (StatSoft. Inc., Tulsa, OK, USA) and differences were always considered significant at values of *P* < 0.05.

## Results

**Fig. 2:**
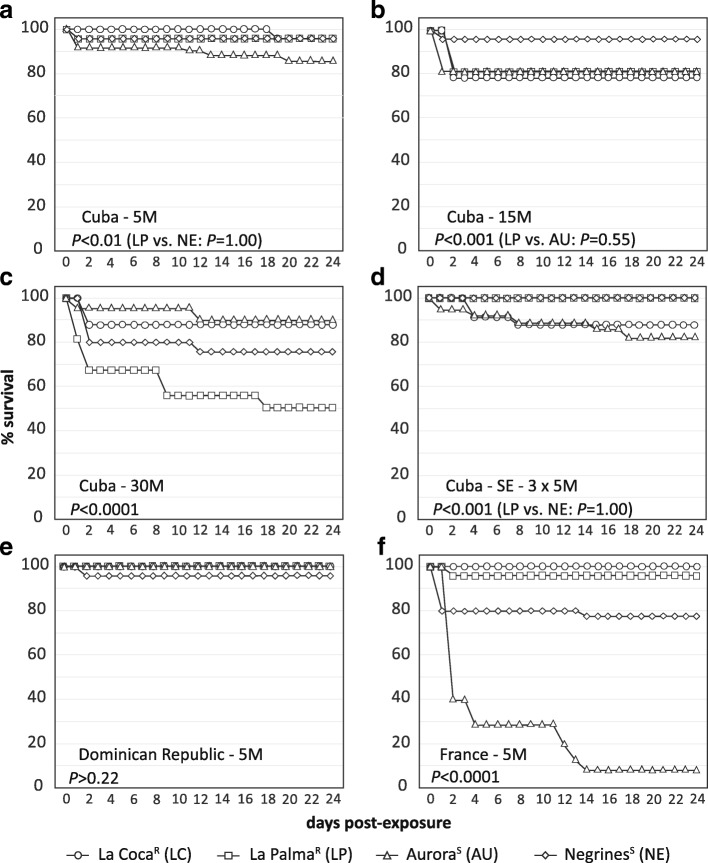
Survival curves of experimentally-exposed resistant (R) and susceptible (S) *Pseudosuccinea columella* to *Fasciola hepatica*. Snails were challenged following different approaches: **a**-**c** increasing miracidial doses, **d** serial exposures (SE) and **e**-**f** exposure to allopatric *F. hepatica* isolates. Pairwise comparisons of snail survival between populations at each experimental trial were performed by log-rank tests and are shown. *Abbreviation*: M, miracidia

Results of host survival in the exposed groups are shown in Fig. 2. Overall, we observed a mortality peak on exposed snails occurring at 24–48 h post-exposure and a significant decrease of snail survival with increasing miracidia dose (Fig. 2a-d; log-rank tests: *P* < 0.05; Aurora, 5 M *vs* 30 M, *P* = 0.24; Negrines, 5 M *vs* 15 M, *P* = 1). No mortality was observed within the studied time frame in the control group (non-exposed snails; data not shown).

### Infection outcome in resistant *P. columella*

As expected, while susceptible *P. columella* became infected after exposure to *F. hepatica*, resistant snails from La Palma and La Coca failed to develop larval stages and no sign of infection was observed following each of the experimental approaches tested (Table 1). Notably, resistant populations showed higher overall survival than susceptible *P. columella* (Fig. 2).

**Table 1:** Prevalence (95% confidence interval) of *Fasciola hepatica* in resistant and susceptible *Pseudosuccinea columella* using different experimental exposure approaches

Experimental infections	Experimental groups	*Pseudosuccinea columella* populations (%)			
		La Coca	La Palma	Aurora	Negrines
		(*n* = 30)^a^	(*n* = 30)^a^	(*n* = 30)^b^	(*n* = 30)^b^
Increasing infective dose: sympatric *F. hepatica*	5 M	0	0	66.7 (48.1–85.2)	63.3 (44.4–82.2)
	15 M	0	0	83.0 (66.4–92.6)	90.0 (74.4–96.5)
	30 M	0	0	93.3 (78.7–98.2)	80.0 (62.7–90.5)
Serial exposure: sympatric *F. hepatica*	3 × 5 M	0	0	90.0 (74.4–96.5)	100 (88.7–100)
Exposure to allopatric *F. hepatica*	Dominican Republic 5 M	0	0	50.0 (33.1–66.9)	66.7 (48.8–80.8)
	France 5 M	0	0	13.3 (4.2–29.9)	60.0 (40.7–76.6)

*Abbreviation*: *M* miracidia

^a^Resistant populations

^b^Susceptible populations

### Infection outcome in susceptible *P. columella*, increasing miracidial dose and serial exposures

**Fig. 3:**
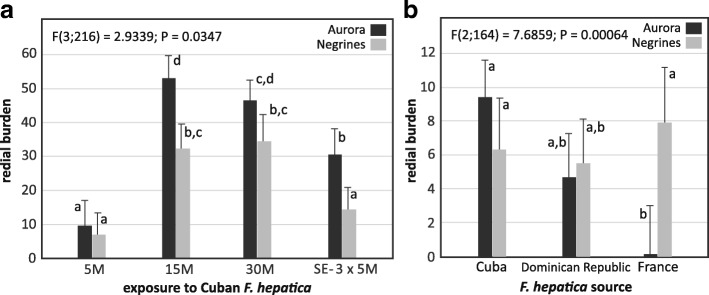
Redial burden of *Fasciola hepatica* in two susceptible *Pseudosuccinea columella* populations using different experimental exposures (factorial ANOVA results). **a** Exposure to different doses of 5, 15 and 30 miracidia (M) and a serial (three times) exposure to 5 miracidia of Cuban *F. hepatica* (SE: 3 × 5 M) **b** Exposure to a dose of five miracidia of sympatric (Cuban) and allopatric *F. hepatica* isolates. Vertical bars denote 95% confidence interval and different letters show differences between means after a *post-hoc* Tukey test

An overall increase of both prevalence (Fisher’s exact test: Aurora, 5 M *vs* 30 M, *P* = 0.021; Negrines, 5 M *vs* 15 M / 3 × 5 M, *P* < 0.03; Table 1) and redial burden (Fig. 3a) was noted in susceptible individuals with the increase of the miracidial dose, either by single or serial exposure events. However, no significant differences in prevalence and redial burden were observed when snails were infected with 15 M or 30 M (Fisher’s exact test: Aurora, 15 M *vs* 30 M, *P* = 0.423; Negrines, 15 M *vs* 30, *P* = 0.472; Fig. 3a).

An interesting result regarding redial burden was observed when comparing infection outcomes after exposures to 15 miracidia, either singly (15 M) or serially-delivered (3 × 5 M; Fig. 3a): it was significantly lower in serially-exposed susceptible snails (Aurora, *P* = 0.0145; Negrines, *P* = 0.0326; Fig. 3a). Contrastingly, a similar parasite prevalence was attained with both conditions (Fisher’s exact test: Aurora, *P* = 0.7065; Negrines, *P* = 0.2372).

### Infection outcome in susceptible *P. columella*, comparing exposure to sympatric and allopatric *F. hepatica* isolates

Infection with a 5 M dose of either Cuban and Dominican isolates produced similar prevalence and redial burden in the two susceptible *P. columella* populations (Table 1, Fig. 3b). However, compared to the Dominican isolate, a significant decrease on snail survivorship was observed with the Cuban parasite (Fig. 2a, e; log-rank tests: *P* < 0.01).

On the other hand, we found significant differences in compatibility between susceptible *P. columella* and the French *F. hepatica* isolate and this variability depended upon the snail-parasite combination (Table 1, Fig. 3b). A poor performance of French *F. hepatica*, in terms of prevalence and redial burden, was recorded when infecting Aurora snails but it appears as compatible as the Cuban isolate with Negrines (Table 1, Fig. 3b). It is noteworthy that an overall impairment of snail survival on susceptible populations was observed with the French parasite isolate when compared with the sympatric *F. hepatica* (Fig. 2f; log-rank tests: *P* < 0.001).

## Discussion

### *P. columella* resistance to *F. hepatica* remains after high parasite doses, serial exposures and parasites from different geographical origins

*Fasciola hepatica* infection of its snail host is a dynamic and complex process in which several stages (i.e. snail-finding, penetration, migration, establishment and larval development) and requirements (e.g. biochemical, physiological and immunological), accounting for host-parasite compatibility, must be met to ensure parasite success [22]. In the present study, the experimental exposure of *P. columella* snails to *F. hepatica* was followed by the successful penetration of all miracidia into the snails in every trial. After this initial interaction, the first 24–48 h are crucial to the final outcome of the infection. In this time frame, a serial of fundamental events occurs inside the host: (i) complete transformation of miracidia into sporocysts [22]; (ii) migration within the digestive tract and first colonization attempts [23]; and (iii) orchestration of snail defence response, whether protective or not [12]. Either compatible *F. hepatica* larva survive and become established (the host becomes infected), or no further parasite development occurs and exposed snails are free from infection. Given the complexity of the initial interaction, it is not surprising that a peak of mortality in *P. columella* occurred during the first 24–48 h post-exposure, even in individuals from resistant populations (see Fig. 2).

As mentioned before, exposure to a large number of miracidia and/or to serial infections could differentially modify the parasite/host possibilities to infect or to be infected, most likely in favour of the parasite [17]. Our results on susceptible *P. columella* coincide with other investigations that have also rendered higher infection rates, mainly in sympatric snail-trematode combinations, after experimental infections with large numbers of parasite or serial miracidial exposures [17, 24, 25]. This strongly points at the pertinence of the first two experimental schemes used for tipping the scales in favour of parasite success. However, in this sense, the effectiveness of the resistant phenotype was not affected even after being challenged with a large number of miracidia and an enhanced genetic diversity of the parasite (either singly or serially delivered) with which each snail was confronted (*F. hepatica* isolate from La Palma isolate is known to be polymorphic [15]). Thus, host involvement in defence contributing to parasite elimination in resistant *P. columella* appears to be enough to protect the snails even from high infective doses without reversion of the resistant phenotype.

In addition, we investigated if resistant *P. columella* snails could resist other parasite isolates beyond the local (Cuban) isolates, challenging them with geographically distant *F. hepatica*, in an attempt to increase the chances of exposing the snails to parasites with different genetic diversity. Although there are no data on the genetic population structure of the allopatric *F. hepatica* isolates used, this species is known to be highly polymorphic with a preferential out-crossing reproduction [26]. Moreover, we should expect genetic differences among the isolates used given the geographical isolation, particularly between the Cuban and the European flukes. In this sense, the differences in *F. hepatica* performance between allopatric and sympatric isolates when infecting susceptible *P. columella* suggest significant differences in compatibility depending on host-parasite combination (as seen elsewhere [14]). These differences, most likely related with the assumed genetic differences, were highly marked between *F. hepatica* from Cuba and France. However, no infection developed in resistant snails exposed to either allopatric *F. hepatica* used, suggesting that the defence mechanisms involved in the resistant phenotype (protective immune response towards the parasite) might not be tightly restricted to a local genotype-genotype type interaction.

The underlying mechanism mediating the encapsulation of *F. hepatica* observed in resistant *P. columella* 24 h post-exposure [12] remains to be fully elucidated. However, mounting an efficient immune response against *F. hepatica*, or any other parasites, is expected to be costly [27] and would certainly results in trade-off against significant energy and resources of the host. However, host investment in defence might result all-too-costly for some individuals, particularly when parasites are highly pathogenic or virulent (as seen for the French *F. hepatica-*Aurora snails interaction), if the infective dose is high, or even if the regulatory mechanisms of the host response are not entirely effective. This may explain the decreased survival of resistant snails when exposed to a high miracidia dose, an effect that was also observed by Gutierrez et al. [12]. In any case, and considering both the individual and population levels, resistant *P. columella* populations failed to develop the infection.

### Redial production in relation to high miracidial doses and successive exposure approaches

As for parasite prevalence, redial burden had an overall increase in susceptible snails when infected with doses higher than 5 M. However, the method of delivering the infective parasite dose influenced the parasite count (more rediae in a single 15 M dose compared to serially-exposed snails). This result could be linked to an increased ability to control parasite development on serially-exposed snails driven by boosting defences with the first exposure, a phenomenon commonly known as immune priming. Immune priming is acknowledged to occur in invertebrates and has been previously reported in the system *Biomphalaria glabrata*-*Schistosoma mansoni*, where enhanced protection of the host (ranging from decreased prevalence and parasite intensity to complete protection) may appear after subsequent parasite encounters [28–30]. According to these authors, the extent of such acquired protection increases towards genetically similar challenges and when re-exposure occurs 10 days after the primary infection. The demonstration of an immunological memory process in susceptible *P. columella*-*F. hepatica* deserves further investigations.

On the other hand, no significant variation was observed in infected snails exposed to 15 or 30 M which could be related to intraspecific competition of parasite larvae for host resources. Rondelaud et al. [31] referred that multiple miracidial infections could impact *F. hepatica* productivity in its intermediate host by limiting redial numbers on second and third generations.

### Performance of allopatric *F. hepatica* with susceptible *P. columella* snails

In a previous study involving experimental infections with several Cuban *F. hepatica-*lymnaeid snail combination, Vázquez et al. [14] proposed the existence of a polymorphism of compatibility in this interaction when presented with a variety of outcomes in terms of snail survival and the parasite’s prevalence and redial burden in susceptible snails. From our results, we also observed differences in compatibility between the local and allopatric *F. hepatica*-*P. columella* systems that varied depending on the host-parasite combinations.

In this sense, the significantly lower performance of French *F. hepatica* in susceptible *P. columella* from Cuba contrasted with what was observed with the Dominican allopatric isolate. The latter displayed a similar prevalence and redial burden to the local Cuban isolate. These results could be related with the peculiarities of the epidemiological scenario from each geographical area (Caribbean and Europe), especially concerning the differences on intermediate host species and environmental conditions. In fact, lymnaeid vectors of fasciolosis in Cuba (*Galba cubensis* and *P. columella*) are the same occurring in the Dominican Republic and in most of the Caribbean region [32, 33]. Thus, even when local adaptation of Dominican parasites to Cuban snail populations is improbable due to geographical isolation (both are separated islands), lower differences can be expected between allopatric parasites evolving under similar conditions (e.g. the same host species) and could explain the similar performances recorded between Cuban and Dominican *F. hepatica*. Conversely, *F. hepatica* transmission in France is mainly related to other snails (e.g. *Galba truncatula* and *Omphiscola glabra*) [34] which could lead to a divergent evolution of the European parasite and may explain its lower compatibility with Cuban *P. columella*. Rondelaud et al. [35] also observed differential redial development and cercarial production when parasites from Argentina and France were tested against a European *G. truncatula*. Thus, different vector species from different geographical regions could be particularly important when analysing compatibility of allopatric and sympatric *F. hepatica* isolates and might bring a deeper understanding on parasite evolution.

## Conclusions

We found that resistance in *P. columella* to *F. hepatica* infection remains independent of the parasite dose, serial parasite exposures or the geographical origin of the parasite. Conversely, the *F. hepatica*-susceptible *P. columella* interaction seems specific for infection and is favoured by high parasite doses. Finally, our results endorse the potential use of resistant *P. columella* snails as an alternative for the control of parasite transmission.

## Abbreviations

M: Miracidia
R: Resistant
S: Susceptible
